# Chemical Constituents and Antifungal Activity of *Ficus hirta* Vahl. Fruits

**DOI:** 10.3390/plants6040044

**Published:** 2017-09-27

**Authors:** Chunpeng Wan, Chuying Chen, Mingxi Li, Youxin Yang, Ming Chen, Jinyin Chen

**Affiliations:** 1 Jiangxi Key Laboratory for Postharvest Technology and Nondestructive Testing of Fruits & Vegetables; Collaborative Innovation Center of Post-Harvest Key Technology and Quality Safety of Fruits and Vegetables; College of Agronomy, Jiangxi Agricultural University, Nanchang 330045, China; chunpengwan@jxau.edu.cn (C.W.); ccy0728@126.com (C.C.); liming.xi@hotmail.com (M.L.); yangyouxin@jxau.edu.cn (Y.Y.); chenming@jxau.edu.cn (M.C.); 2 Pingxiang University, Pingxiang 337055, China

**Keywords:** *Ficus hirta*, Moraceae, carboline alkaloids, sesquiterpenoids, flavonoids, antifungal

## Abstract

Phytochemical investigation of *Ficus hirta* Vahl. (Moraceae) fruits led to isolate two carboline alkaloids (**1** and **2**), five sesquiterpenoids/norsesquiterpenoids (**3–7**), three flavonoids (**8–10**), and one phenylpropane-1,2-diol (**11**). Their structures were elucidated by the analysis of their 1D and 2D NMR, and HR-ESI-MS data. All of the isolates were isolated from this species for the first time, while compounds **2**, **4–6**, and **8–11** were firstly reported from the genus *Ficus*. Antifungal assay revealed that compound **8** (namely pinocembrin-7-*O*-β-d-glucoside), a major flavonoid compound present in the ethanol extract of *F. hirta* fruits, showed good antifungal activity against *Penicillium italicum*, the phytopathogen of citrus blue mold caused the majority rotten of citrus fruits.

## 1. Introduction

The genus *Ficus* (Moraceae) contains more than 1000 species, most of them are distributed in tropical, sub-tropical, and Mediterranean regions [1]. There are around 98 species distributed in the South of China. *Ficus hirta* Vahl. is mainly distributed in Yunnan, Guizhou, Guangxi, Guangdong and Hainan province, China [1]. The fruits of *F. hirta* were used as medicine and food resource by the local people of Guangdong province, China. Previous chemical investigations on the *F. hirta* focused on its roots, which led to isolate and identify the predominant chemical constituents, flavonoids, and coumarins. To date, the total of 31 flavonoids [2,3,4,5,6,7] and 7 coumarins [2,3,5,8] have been reported from this species. Except the flavonoids and coumarins, there are some other compounds reported from this species, such as steroids [2,7] and benzoic acid derivatives [7]. 

Several studies on the pharmacological activities of *F. hirta* showed its antioxidation [9], cytotoxicity, and apoptosis of HeLa cells [10]; anti-inflammation and analgesia [11], antitussive and antiasthmatic [12], hepatoprotective [13], and radioresistance effects [14]. The fruits of *F. hirta* consumed as a plant-derived food that showed potential tonic effects [15]. Besides mentioned above, *F. hirta* also showed antibacterial activity against *Escherichia coli*, *Staphylococcus aureus* [16], and *Penicillium italicum*, a phytopathogenic cause of citrus blue mold resulted in the destructive fruit rotten of citrus. The fruits of *F. hirta* and several other medicinal plants were also used to control the phytopathogen in order to decrease the loss of citrus rotten [17,18]. The fruits of *F. hirta* showed promising antifungal activity against *P. italicum* and prolonged the Nanfeng mandarin preservation period [19], while the major antifungal constituents were not clear until now. 

In order to continue our studies on isolation and identification of the antifungal compounds from plants. We have elucidated the antifungal constituents of *F. hirta* fruits. Fortunately, our previous studies have identified nine monosubstituted benzene derivatives from the extracts of *F. hirta* fruits and some of them showed good antifungal activities [17,18], while they were not the major antifungal constituents for their low content in the plant. In continuation, the current study was aimed to discover the major antifungal compounds with diverse structures from this species.

## 2. Results

As described previously, the ethanol extracts (FH) of the fruits of *F. hirta* and the fractions (FH1–FH4) fractionated by D101 macro resin column were evaluated for their antifungal activities against *P. italicum*. Fractions FH2–FH4 showed stronger antifungal activities in a concentration-dependent manner than that of FH crude extract [18]. The further isolation was focused on the fractions with potent antifungal activities to find more active compounds present in the fruits of *F. hirta*. 

**Figure 1:**
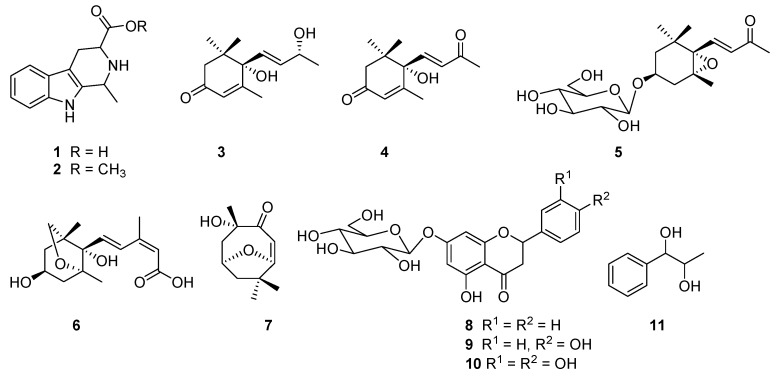
Structures of the compounds isolated from the fruits of *Ficus hirta*.

As a result, 11 compounds (**1–11**) (Figure 1) were isolated from those fractions, and their structures were elucidated based on the analysis of spectroscopic data (including HR-ESI-MS, ^1^H-NMR, ^13^C-NMR, and 2D NMR) and comparison of these data to previous published paper. The 11 compounds were determined as 1-methyl-1,2,3,4-tetrahydro-β-carboline-3-carboxylic acid (**1**) [20], methyl 1-methyl-1,2,3,4-tetrahydro-β-carboline-3-carboxylate (**2**) [21], vomifoliol (**3**) [22], dehydrovomifoliol (**4**) [22], icariside B_2_ (**5**) [23], dihydrophaseic acid (**6**) [24], pubinernoid A (**7**) [25], pinocembrin-7-*O*-β-d-glucoside (**8**) [26], naringenin-7-*O*-β-d-glucoside (**9**) [27], eriodictyol-7-*O*-β-d-glucoside (**10**) [28], and 1-phenylpropane-1,2-diol (**11**) [29]. All of the isolates were isolated from this species for the first time, while compounds **2**, **4**–**6**, and **8**–**11** were first reported from the genus *Ficus*. 

**Figure 2:**
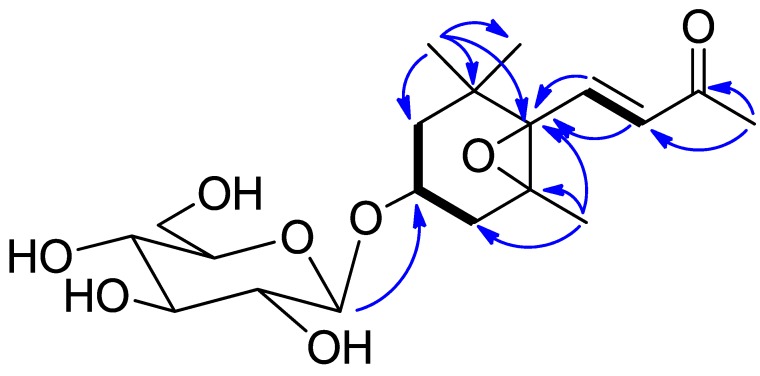
^1^H–^1^H COSY (bold bonds) and HMBC (arrows) correlations of Compound **5**.

Compound **5**, was obtained as colorless amorphous solid, displayed a molecular formula of C_1__9_H_30_O_8_ as determined by HRESIMS at *m*/*z* 409.1822 [M + Na]^+^ (calcd. for C_1__9_H_30_O_8_Na, 409.1838). In the ^1^H-NMR spectrum, four tertiary methyls signals (δ_H_ 2.29, 1.21, 1.19, 0.96, each 3H, s), a *trans* double bond protons signal at δ_H_ 7.17 (1H, d, *J* = 15.8 Hz) and 6.18 (1H, d, *J* = 15.8 Hz), and an anomeric proton at δ_H_ 4.34 (1H, d, *J* = 7.0 Hz), as well as oxygen-bearing methine protons at δ_H_ 3.86 (1H, m) were observed. The ^13^C-NMR and HSQC spectra revealed the presence of 19 carbon resonances, 6 of them were contributed to glucose. Analysis of the 1D and 2D NMR spectra data (including ^1^H–^1^H COSY, HSQC, HMBC) allowed for the establishment of the structure of **5**. The HSQC spectrum permitted the assignment of all of the protons to their bonding carbons. The ^1^H–^1^H COSY spectra (drawn with bold bonds in Figure 2) disclosed that compound **5** had three partial structure units (including a sugar moiety). Analysis of the HMBC spectrum then enabled the connectivity of these spin coupling fragments and the other functional groups. The HMBC correlations (Figure 2) from H_3_-12 (H_3_-13) to C-1, C-2, C-6, and C-13 (C-12); from H-11 to C-4, C-5, and C-6; from H-7 and H-8 to C-6; and from H_3_-10 to C-8 and C-9, allowed the construction of the planar structure of aglycone. The glucopyranose was linked to C-3 by the HMBC correlation from H-1′ to C-3. Searching the structure with SCIFINDER revealed it has the same planar structure as the NMR data of **5** with those of icariside B_2_ indicated they had the same stereochemistry. Therefore, compound **5** was determined as icariside B_2_. 

**Figure 3:**
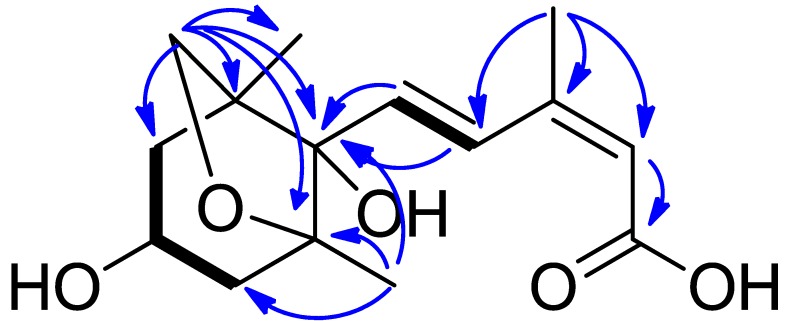
^1^H–^1^H COSY (bold bonds) and HMBC (arrows) correlations of Compound **6**.

Compound **6**, had a molecular formula of C_15_H_2__2_O_5_ as determined by HRESIMS at *m*/*z* 305.1349 [M + Na]^+^ (calcd. for C_15_H_2__2_O_5_Na, 305.1365). The ^1^H-NMR spectrum showed a *trans* double bond signals at δ_H_ 7.98 (1H, d, *J* = 15.8 Hz) and 6.52 (1H, d, *J* = 15.8 Hz), an olefinic proton signal at δ_H_ 5.76 (1H, s), an oxygenated methylene signal at δ_H_ 3.80 (1H, d, *J* = 7.4 Hz) and 3.70 (1H, d, *J* = 7.4 Hz), and three tertiary methyls signals (δ_H_ 2.08, 1.15, 0.93, each 3H, s). The ^13^C-NMR and HSQC spectra revealed the presence of 15 carbon signals, attributing to 3 methyls, 3 methylenes, 4 methines, and 5 quaternary carbons. Aforementioned data suggested that compound **6** was likely a sesquiterpenoid. Further analysis of 2D NMR (^1^H–^1^H COSY, HSQC, and HMBC) data allowed us to determine the structure of **6**. The ^1^H–^1^H COSY correlations of H-2/H-3, H-3/H-4, and H-7/H-8 indicated the presence of two structure units (drawn with bold bonds in Figure 3). The HMBC correlations (Figure 3) from H_2_-12 to C-1, C-2, C-5, C-6, and C-13; from H_3_-14 to C-4, C-5, and C-6; from H-7 and H-8 to C-6; from H_3_-15 to C-8, C-9 and C-10; and from H-10 to C-11, constructed the planar structure of **6**. Compared the NMR data of **6** with those of dihydrophaseic acid revealed they had the same structure. Therefore, compound **6** was elucidated as dihydrophaseic acid.

The antifungal activities of all of the isolates were tested at two concentrations (2.0 and 4.0 mg/mL). The results showed that none of them except compound **8** showed antifungal activity with the DIZs of 19.0 ± 0.5 mm and 24.0 ± 0.5 mm at 2.0 and 4.0 mg/mL, respectively, which are more powerful than that of FH (11.0 ± 0.6 mm at 2.0 mg/mL).

Moreover, compound **8** was also evaluated, its antifungal activity using mycelia growth method, the results are shown in Table 1. The inhibition rate was shown as concentration-dependent. Compound **8** exhibited more than 90% inhibitory effect against *P. italicum* at 400 µg/mL, while 100% inhibition rate was achieved at 800 µg/mL.

## 3. Discussion

The genus *Ficus* is characterized by flavonoids, coumarins, terpenoids (triterpenoids and sesquiterpenoids), and alkaloids [30]. In the current study, 11 compounds (**1**–**11**) were isolated and identified from the fruits of *F. hirta*, which were classified as carboline alkaloids (**1** and **2**), sesquiterpenoids/norsesquiterpenoids (**3**–**7**) and flavonoid glucosides (**8**–**10**). The structural classes of these isolates support the taxonomic placement of *F. hirta* in the genus *Ficus*. All of the chemical constituents are isolated from this species for the first time. Moreover, compounds **2**, **4**–**6**, and **8**–**11** are firstly reported from the genus *Ficus*. Compound **1** has been isolated from *Ficus pumila* [17], which is the only carboline alkaloid reported from the genus *Ficus* before the current study. Five sesquiterpenoids/norsesquiterpenoids (**3**–**7**) could be further classified as megastigmanes (**3** and **5**), carotenoid sesquiterpenoid (**6**), and norsesquiterpenoid with 11C skeleton (**7**). Vomifoliol (**3**) has been exclusively obtained from the species of *Ficus platypoda* and *Ficus pumila* before our study [17,31]. 

The discovery of compounds **1** and **3** showed the relevance between this species and other *Ficus* species such as *F. platypoda* and *F. pumila*. Compound **6** is a derivative of plant hormone abscisic acid that can be classified as carotenoid sesquiterpenoid, which is different from other sesquiterpenoids isolated from the genus *Ficus* [32,33]. Compound **7** is a norsesquiterpenoids with 11C skeleton with different linkage with other 11C skeleton norsesquiterpenoids previously isolated from *Ficus microcarpa* [34,35]. Previously, two isomers of “Eriodictyol hexoside” and other flavonoids were tentative identified in the fruits of *F. carica* using HPLC-MS approaches [36]. The identification of eriodictyol-7-*O*-β-d-glucoside (**10**) in *F. hirta* confirmed these results and showed the relevance between this species and *F. carica*.

Pinocembrin-7-*O*-β-d-glucoside (**8**) showed good antifungal activity while the compounds **9** and **10** showed none activity**,** which indicated the antifungal activity of flavonoids maybe effected by the number of hydroxyl in the C loop. This is the first time the antifungal activity of compound **8** against *P. italicum* has been reported. However, some references have already revealed the antifungal activity of its aglycone, namely pinocembrin [37,38].

Overall, these results indicated that carboline alkaloid, megastigmanes, and flavonoids could be regarded as a chemotaxonomic marker of *F. hirta*. Also, while only one carotenoid sesquiterpenoid and a norsesquiterpenoid with 11C skeleton were identified herein, whether they may be regarded as a chemotaxonomic marker of *F. hirta* species remains to be established. Pinocembrin-7-*O*-β-d-glucoside (**8**) was the major antifungal constituent against *P. italicum* existed in the fruits of *Ficus hirta*.

## 4. Materials and Methods

### 4.1. Plant Material

The fruits of *F. hirta* were bought from Zhangshu medicinal market, Jiangxi Province, China, and authenticated by Prof. Shouran Zhou (College of Basic Medicine, Jiangxi University of Traditional Chinese Medicine). A voucher specimen (no. FH-201406) was deposited in the herbarium of Jiangxi Key Laboratory for Postharvest Technology and Nondestructive Testing of Fruits & Vegetables, Jiangxi Agricultural University (Nanchang, Jiangxi, China).

### 4.2. Equipment and Reagents

^1^H- and ^13^C-NMR spectral data were tested on a Varian 400 MHz Nuclear magnetic resonance spectrometer with Tetramethylsilane (TMS) as internal standard. HR-ESI-MS were detected on a TripleTOF™ 5600 LC/MS/MS (Applied Biosystems MDS, Foster City, CA, USA) mass spectrometer. Medium pressure liquid chromatography (MPLC) was carried out on a C-605 pump (BUCHI, Flawil, Switzerland) coupled with a reverse phase C18 column (3.6 × 46 cm). HPLC was conducted on a Hitachi Elite Chromaster system—consisting of a 5210 autosampler, 5110 pump, 5430 diode array detector, and 5310 column oven—which were operated by EZChrom Elite software. Luna C18 column (5 µm, 4.6 × 250 mm) for analysis and Luna C18 column (5 µm, 10 × 250 mm) for semi-preparative HPLC were purchased from Phenomenex Inc. (Torrance, CA, USA). The HPLC grade solvents were purchased from Sigma (Sigma, St. Louis, MO, USA). All analytical solvents were bought from Tansoole (Shanghai, China).

### 4.3. Extraction and Chromatography

The air dried fruits of *F. hirta* (4.9 kg) were ground and extracted using ultrasonic-assisted method with 95% ethanol (3 × 25 L) at 25 °C for 90 min. The extract were evaporated to remove ethanol solvent and yielded the dried ethanol extract (345.1 g), which was subjected to D101 macro rein column chromatography eluted with water, 30% ethanol (*v*/*v*), 50% ethanol, and 95% ethanol, respectively, to yield four fractions (FH1–FH4). Antifungal activity test indicated that three fractions (FH2–FH4) were the active fractions. Activity-guided isolation were performed accordingly, as follows.

The 30% ethanol fraction FH2 (113.6 g) was subjected to C_18_ silica gel column (3.6 × 46 cm) chromatography elution with MeOH/H_2_O (MeOH/H_2_O, 15/85 to 25/75, *v*/*v*) to yield five fractions (FH2a–FH2e). Fraction FH2c was separated on Sephadex LH-20 and eluted with MeOH to give six combined sub-fractions (FH2c1–FH2c6). Fraction FH2c2 was subjected to Sephadex LH-20 elution with MeOH to give five sub-fractions (FH2c2a–FH2c2e). Sub-fraction FH2c2c was purified using silica gel column chromatography using CH_3_Cl-MeOH (10:1 to 1:1, *v*/*v*) for elution to give compound **1** (12.0 mg) and two sub-fractions (FH2c2c1 and FH2c2c2). Purification of FH2c2c1 with semi-preparative HPLC, eluting with MeOH-H_2_O (0–25 min: 20:80 to 55:45; 25–26 min: 55:45 to 100:0; 26–27 min: 100:0; 27–28 min: 100:0 to 20:80; 28–35 min: 20:80; *v*/*v*, 3 mL/min), yielded compound **5** (2.4 mg). Fraction FH2c2d was subjected to silica gel column chromatography eluted with CH_3_Cl-MeOH (100:1 to 1:1, *v*/*v*) to get seven fractions (FH2c2d1–FH2c2d7). 

Purification of fraction FH-2C2d1 with semi-preparation HPLC, eluting with MeOH-H_2_O (0–25 min: 20:80 to 68:32; 25–26 min: 68:32 to 100:0; 26–27 min: 100:0; 27–28 min: 100:0 to 30:70; 28–35 min: 30:70; *v*/*v*, 3 mL/min), yielded compound **4** (3.6 mg).

Fraction FH2c2d2 was purified by semi-preparative HPLC, eluting with MeOH-H_2_O (0–20 min: 20:80 to 51:49; 20–21 min: 51:49 to 100:0; 21–22 min: 100:0; 22–23 min: 100:0 to 20:80; 23–30 min: 20:80; *v*/*v*, 3 mL/min), yielded compounds **7** (4.0 mg) and **11** (4.5 mg). Fraction FH2c2d5 was subjected on Sephadex LH-20 eluted with MeOH to give three sub-fractions (FH2c2d5a–FH2c2d5c). Purification of FH2c2d5b with semi-preparative HPLC, eluting with MeOH-H_2_O (0–35 min: 30:70 to 34:66; 35–36 min: 34:66 to 100:0; 36–37 min: 100:0; 37–38 min: 100:0 to 30:70; 38–45 min: 30:70; *v*/*v*, 3 mL/min), yielded compounds **2** (3.2 mg) and **6** (8.2 mg). Fraction FH2c3 was purified by semi-preparative HPLC, eluting with MeOH-H_2_O (0–21 min: 20:80 to 55:45; 21–22 min: 55:45 to 100:0; 22–23 min: 100:0; 23–24 min: 100:0 to 20:80; 24–31 min: 20:80; *v*/*v*, 3 mL/min), yielded compound **3** (9.7 mg). Fraction FH2d was separated on Sephadex LH-20 eluted with MeOH to give eleven combined sub-fractions (FH2d1–FH2d11). Fraction FH2d6 was recrystallized with methanol to yield compound **10** (15.4 mg). 

The 50% ethanol fraction FH3 (35.5 g) was subjected to C_18_ silica gel column chromatography eluted with MeOH/H_2_O (MeOH/H_2_O, 40/60 to 70/30, *v*/*v*) to yield six fractions FH3a–FH3f. Fraction FH3c was separated on Sephadex LH-20 eluted with MeOH to give eight combined sub-fractions (FH3c1–FH3c8). Fraction FH3c7 was recrystallized with methanol to yield compound **9** (13.2 mg).

The 95% ethanol eluted fraction FH4 (7.2 g) was separated over a column of Sephadex LH-20 eluted with MeOH to give eight combined sub-fractions (FH4a–FH4h). Fraction FH4e was recrystallized with methanol to yield compound **8** (21.5 mg).

### 4.4. Antifungal Activity Test

The antifungal activity of FH extracts and isolates against *P. italicum* was evaluated by the Oxford Cup method as described previously [17,18].

The antifungal activity of the pure compound **8** (pinocembrin-7-*O*-β-d-glucoside) against *P. italicum* were further examined by the mycelia growth method as described previously [17]. Briefly, the pure compound **8** were dissolved in 95% ethanol, and then added to the sterile PDA (potato dextrose agar) culture medium at the specified concentrations. The mixed media were then poured into plastic Petri dishes (90 mm). The agar-mycelial plugs (6 mm) infected with pathogens were incubated at the center of the Petri dishes sealed with parafilm and incubated in the dark. Mycelium colony growth diameters were measured when the fungal mycelium of the control group had completely covered the Petri dishes. All treatments were tested in six replicates. The inhibition of mycelial growth (IMG, %) was calculated as the following formula: IMG (%) = 100 × (dc − dt)/(dc − 6), where dc and dt were the mycelium diameters (mm) of the control and the treatment, respectively.

### 4.5. NMR and MS Data of Compounds ***1***–***11***

The ^1^H- and ^13^C-NMR data of these compounds (**1***–***11**) were listed as follows.

*Compound* **1** HR-ESI-MS *m*/*z* 231.1130 [M + H]^+^, ^1^H-NMR (400 MHz, DMSO-*d*_6_) δ: 4.52 (1, H d, *J* = 6.0 Hz, H-1), 3.64 (1H, dd, *J* = 4.5, 12.0 Hz, H-3), 2.78 (1H, m, H-4b), 7.45 (1H, d, *J* = 7.8 Hz, H-5), 7.01 (1H, t, *J* = 7.2 Hz, H-6), 7.10 (1H, t, *J* = 7.2 Hz, H-7), 7.34 (1H, d, *J* = 7.8 Hz, H-8), 3.18 (1H, dd, *J* = 4.5, 15.3 Hz, H-4a), 1.63 (3H, d, *J* = 6.2 Hz, CH_3_). ^13^C-NMR (100 MHz, DMSO-*d*_6_) δ: 49.5 (C-1), 58.1 (C-3), 23.7 (C-4), 118.5 (C-5), 119.3 (C-6), 121.8 (C-7), 111.7 (C-8), 107.2 (C-4a), 126.6 (C-4b), 136.8 (C-8a), 132.7 (C-9), 17.4 (C-10), 170.0 (C-11).

*Compound* **2** HR-ESI-MS *m*/*z* 245.1284 [M + H]^+^, ^1^H-NMR (400 MHz, CD_3_OD) δ: 4.60 (1H, dd, *J* = 5.3, 12.0 Hz, H-1), 3.95 (3H, s, OCH_3_), 3.59 (1H, dd, *J* = 4.8, 11.2 Hz, H-3), 3.52 (1H, t, *J* = 6.0 Hz, H-4b), 7.50 (1H, d, *J* = 7.8 Hz, H-5), 7.07 (1H, t, *J* = 7.2 Hz, H-6), 7.16 (1H, t, *J* = 7.2 Hz, H-7), 7.37 (1H, d, *J* = 7.8 Hz, H-8), 3.14 (1H, m, H-4b), 1.78 (3H, d, *J* = 6.2 Hz, CH_3_). ^13^C-NMR (100 MHz, CD_3_OD) δ: 50.2 (C-1), 55.6 (C-3), 22.3 (C-4), 117.7 (C-5), 119.4 (C-6), 122.3 (C-7), 110.0 (C-8), 104.6 (C-4a), 125.7 (C-4b), 137.2 (C-8a), 129.4 (C-9), 15.6 (C-10), 169.0 (C-11), 52.5 (OCH_3_).

*Compound* **3** HR-ESI-MS *m*/*z* 247.1291 [M + Na]^+^, ^1^H-NMR (400 MHz, CD_3_OD) δ: 1.00 (3H, s, CH_3_-11), 1.03 (3H, s, CH_3_-12), 1.24 (3H, d, *J* = 6.8 Hz, CH_3_-10), 1.91 (3H, d, *J* = 1.2 Hz, CH_3_-13), 2.16 (1H, d, *J* = 16.4 Hz, H-2a), 2.48 (1H, d, *J* =16.4 Hz, H-2b), 4.32 (1H, dq, *J* = 6.4, 6.4 Hz, H-9), 5.78 (1H, d, *J* = 16.0 Hz, H-7), 5.80 (1H, dd, *J =* 16.0, 6.4 Hz, H-8), 5.87 (1H, q, *J* = 1.4 Hz, H-4). ^13^C-NMR (100 MHz, CD_3_OD) δ: 41.0 (C-1), 49.3 (C-2), 199.8 (C-3), 125.7 (C-4), 166.0 (C-5), 79.1 (C-6), 128.5 (C-7), 135.5 (C-8), 67.2 (C-9), 18.1 (C-10), 23.1 (C-11), 22.4 (C-12), 22.0 (C-13).

*Compound* **4** HR-ESI-MS *m*/*z* 221.1164 [M − H]^−^, ^1^H-NMR (400 MHz, CD_3_OD) δ: 1.01 (3H, s, CH_3_-11), 1.06 (3H, s, CH_3_-12), 2.31 (3H, s, CH_3_-10), 1.90 (3H, d, *J* = 1.2 Hz, CH_3_-13), 2.31 (1H, d, *J* = 16.4 Hz, H-2a), 2.62 (1H, d, *J* = 16.4 Hz, H-2b), 6.98 (1H, d, *J* = 16.0 Hz, H-7), 6.43 (1H, dd, *J =* 16.0, 6.4 Hz, H-8), 5.93 (1H, s, H-4). ^13^C-NMR (100 MHz, CD_3_OD) δ: 40.6 (C-1), 49.1 (C-2), 189.9 (C-3), 126.6 (C-4), 163.2 (C-5), 78.6 (C-6), 146.9 (C-7), 130.3 (C-8), 199.2 (C-9), 26.2 (C-10), 22.1 (C-11), 23.3 (C-12), 17.7 (C-13).

*Compound* **5** HR-ESI-MS *m*/*z* 409.1822 [M + Na]^+^, ^1^H-NMR (CD_3_OD, 400 MHz) δ: 7.17 (1H, d, *J* = 15.8 Hz, H-7), 6.18 (1H, d, *J* = 15.8 Hz, H-8), 4.34 (1H, d, *J* = 7.0 Hz, H-1′), 3.86 (1H, m, H-3), 3.12-3.83 (6H, Sugar H-2′, 3′, 4′, 5′, 6′), 2.42 (1H, m, H-4), 2.29 (3H, s, H-10), 1.81 (1H, dd, *J* = 8.2, 14.6 Hz, H-4), 1.73 (1H, m, H-2), 1.40 (1H, m, H-2), 1.21 (3H, s, H-13), 1.19 (3H, s, H-12), 0.96 (3H, s, H-11). ^13^C-NMR (CD_3_OD, 100 MHz) δ: 34.5 (C-1), 43.8 (C-2), 71.3 (C-3), 36.7 (C-4), 66.9 (C-5), 69.7 (C-6), 143.8 (C-7), 132.4 (C-8), 198.8 (C-9), 24.0 (C-10), 26.0 (C-11), 28.0 (C-12), 18.8 (C-13), 101.5 (C-1′), 73.7 (C-2′), 76.7 (C-3′), 70.2 (C-4′), 76.4 (C-5′), 61.3 (C-6′).

*Compound* **6** HR-ESI-MS *m*/*z* 305.1349 [M + Na]^+^, ^1^H-NMR (CD_3_OD, 400 MHz) δ: 7.98 (1H, d, *J* = 15.8 Hz, H-4), 6.52 (1H, d, *J* = 15.8 Hz, H-5), 5.76 (1H, s, H-2), 0.93 (3H, s, H-13), 1.15 (3H, s, H-14), 2.08 (3H, s, H-15), 1.66 (1H, m, H-10b), 1.73 (1H, m, H-8b), 1.84 (1H, m, H-10a), 2.03 (1H, m, H-8a), 3.70 (1H, d, *J* = 7.4 Hz, H-12a), 3.80 (1H, d, *J* = 7.4 Hz, H-12b), 4.11 (1H, m, H-9). ^13^C-NMR (CD_3_OD, 100 MHz) δ: 168.1 (C-1), 117.8 (C-2), 150.1 (C-3), 130.4 (C-4), 133.8 (C-5), 81.8 (C-6), 86.3 (C-7), 44.6 (C-8), 64.6 (C-9), 43.1 (C-10), 75.8 (C-12), 14.9 (C-13), 18.2 (C-14), 19.8 (C-15).

*Compound* **7** HR-ESI-MS *m*/*z* 197.1155 [M + H]^+^, ^1^H-NMR (CD_3_OD, 400 MHz) δ: 5.78 (1H, s, H-6), 4.10 (1H, m, H-2), 2.46 (1H, m, H-3β), 1.98 (1H, m, H-1β), 1.29 (1H, overlap, H-3α), 1.42 (1H, t, *J* = 11.6 Hz, H-1α), 1.59 (3H, s, H-11), 1.31 (3H, s, H-9), 1.28 (3H, s, H-10). ^13^C-NMR (CD_3_OD, 100 MHz) δ: 49.3 (C-1), 63.8 (C-2), 47.6 (overlap, C-3), 87.1 (C-4), 182.4 (C-5), 112.3 (C-6), 172.5 (C-7), 34.7 (C-8), 23.9 (C-9), 28.9 (C-10), 24.3 (C-11).

*Compound* **8** ESI-MS *m*/*z* 417.00 [M − H]^−^, ^1^H-NMR (600 MHz, DMSO-*d*_6_) δ: 12.05 (5-OH), 7.55 (2H, d, *J* = 7.6 Hz, H-2′, 6′), 7.44 (3H, m, H-3′, 4′, 5′), 6.21 (1H, d, *J* = 1.5 Hz, H-8), 6.16 (1H, d, *J* = 1.5 Hz, H-6), 5.66 (1H, d, *J* = 12.9, H-2), 4.99 (1H, d, *J* = 7.4 Hz, H-1″), 3.66 (1H, d, *J* = 9.4 Hz, H-6″a), 3.15-3.45 (6H, H-3a, 2″, 3″, 4″, 5″, 6″b), 2.85 (1H, d, *J* = 16.7 Hz, H-3b). ^13^C-NMR (150 MHz, DMSO-*d*_6_) δ: 79.1 (C-2), 42.6 (C-3), 197.3 (C-4), 163.4 (C-5), 97.1 (C-6), 165.8 (C-7), 96.0 (C-8), 163.0 (C-9), 103.7 (C-10), 138.9 (C-1′), 127.2 (C-2′), 129.1 (C-3′), 129.1 (C-4′), 129.1 (C-5′), 127.2 (C-6′), 100.0 (C-1″), 73.5 (C-2″), 76.8 (C-3″), 69.9 (C-4″), 77.6 (C-5″), 61.0 (C-6″).

*Compound* **9** ESI-MS *m*/*z* 432.90 [M − H]^−^, ^1^H-NMR (600 MHz, DMSO-*d*_6_) δ: 12.06 (5-OH), 7.33 (2H, d, *J* = 7.8 Hz, H-2′, 6′), 6.80 (3H, m, H-3′, 5′), 6.16 (1H, d, *J* = 1.5 Hz, H-8), 6.14 (1H, d, *J* = 1.5 Hz, H-6), 5.50 (1H, d, *J* = 12.7, H-2), 4.96 (1H, d, *J* = 7.6 Hz, H-1″), 3.67 (1H, d, *J* = 9.4 Hz, H-6″a), 3.14-3.46 (6H, H-3a, 2″, 3″, 4″, 5″, 6″b), 2.74 (1H, d, *J* = 16.9 Hz, H-3b). ^13^C-NMR (150 MHz, DMSO-*d*_6_) δ: 79.1 (C-2), 42.5 (C-3), 197.7 (C-4), 163.4 (C-5), 97.0 (C-6), 165.8 (C-7), 95.9 (C-8), 163.2 (C-9), 103.7 (C-10), 129.1 (C-1′), 128.9 (C-2′), 115.7 (C-3′), 158.3 (C-4′), 115.7 (C-5′), 128.9 (C-6′), 100.0 (C-1″), 73.5 (C-2″), 76.8 (C-3″), 69.9 (C-4″), 77.5 (C-5″), 61.0 (C-6″).

*Compound* **10** ESI-MS *m*/*z* 449.10 [M − H]^−^, ^1^H-NMR (600 MHz, CD_3_OD) δ: 6.94 (1H, brs, H-5′), 6.80 (2H, brs, H-2′, 6′), 6.22 (1H, brs, H-8), 6.19 (1H, brs, H-6), 5.32 (1H, d, *J* = 12.6, H-2), 4.98 (1H, d, *J* = 7.4 Hz, H-1″), 3.89 (1H, d, *J* = 12.0 Hz, H-6″a), 3.70 (1H, dd, *J* = 5.3, 12.1 Hz, H-6″b), 3.40–3.48 (4H, H-3a, 2″, 3″, 4″), 3.13 (1H, m, H-5″), 2.75 (1H, d, *J* = 17.0 Hz, H-3b). ^13^C-NMR (150 MHz, CD_3_OD) δ: 79.3 (C-2), 42.8 (C-3), 197.1 (C-4), 163.5 (C-5), 96.6 (C-6), 165.6 (C-7), 95.5 (C-8), 163.2 (C-9), 103.5 (C-10), 130.1 (C-1′), 113.4 (C-2′), 145.1 (C-3′), 145.6 (C-4′), 114.9 (C-5′), 118.0 (C-6′), 99.8 (C-1″), 73.2 (C-2″), 76.4 (C-3″), 69.7 (C-4″), 76.8 (C-5″), 60.9 (C-6″).

*Compound* **11** HR-ESI-MS *m*/*z* 175.0713 [M + Na]^+^, ^1^H-NMR (CD_3_OD, 400 MHz) δ: 7.26–7.35 (5H, m, H-2,3,4,5,6), 4.34 (1H, d, *J* = 7.1 Hz, H-7), 3.80 (1H, m, H-8), 0.95 (3H, d, *J* = 6.4 Hz, H-9). 

## 5. Conclusions

Two carboline alkaloids (**1** and **2**), five sesquiterpenoids/norsesquiterpenoids (**3**–**7**) (three of which are megastigmanes), three flavonoids (**8–10**), and phenylpropane-1,2-diol (**11**) were isolated and identified from the fruits of *F. hirta* for the first time. Moreover, compounds **2**, **4**–**6**, and **8**–**11** were reported for the first time in the *Ficus* genus. Pinocembrin-7-*O*-β-d-glucoside (**8**) was the major antifungal constituent against *P. italicum* existed in the fruits of *Ficus hirta*. Chemotaxonomic analysis revealed that the carboline alkaloid, megastigmanes, and flavonoids could be regarded as a chemotaxonomic marker of *F. hirta*.

## Figures and Tables

**Table 1:** Antifungal activity of Compound **8** tested by mycelia growth method

Concentration (µg/mL)	Inhibition Rate
25	13.70 ± 1.81
50	36.88 ± 1.08
100	56.10 ± 2.55
200	74.65 ± 1.61
400	92.05 ± 1.55
800	100
